# Association of central sensitization, visceral fat, and surgical outcomes in lumbar spinal stenosis

**DOI:** 10.1186/s13018-023-04376-2

**Published:** 2023-11-21

**Authors:** Izaya Ogon, Hiroyuki Takashima, Tomonori Morita, Ryunosuke Fukushi, Tsuneo Takebayashi, Atsushi Teramoto

**Affiliations:** 1 Department of Orthopaedic Surgery, Sapporo Medical University School of Medicine, 291, South-1, West-16, Chuo-ku, Sapporo, 060-8543 Japan; 2 Faculty of Health Sciences, Hokkaido University, North-12, West-5, Kitaku, Sapporo, 060-0812 Japan; 3 Department of Orthopaedic Surgery, Sapporo Maruyama Orthopaedic Hospital, 1-3, North-7, West-27, Chuo-ku, Sapporo, 060-0007 Japan

**Keywords:** Lumber spinal stenosis, Central sensitization, Central sensitization inventory, Visceral fat

## Abstract

**Background:**

Controversy remains regarding predictors of surgical outcomes for patients with lumbar spinal stenosis (LSS). Pain sensitization may be an underlying mechanism contributing to LSS surgical outcomes. Further, obesity is associated with dissatisfaction and poorer outcomes after surgery for LSS. Therefore, this study aimed to examine the relationship between central sensitization (CS), visceral fat, and surgical outcomes in LSS.

**Methods:**

Patients with LSS were categorized based on their central sensitization inventory (CSI) scores into low- (CSI < 40) and high- (CSI ≥ 40) CSI subgroups. The participants completed clinical outcome assessments preoperatively and 12 months postoperatively.

**Results:**

Overall, 60 patients were enrolled in the study (28 men, 32 women; mean age: 62.1 ± 2.8 years). The high-CSI group had significantly higher mean low back pain (LBP), leg pain, and leg numbness visual analogue scale (VAS) scores than the low-CSI group (*p* < 0.01). The high-CSI group had a significantly higher mean visceral fat area than the low-CSI group (*p* < 0.01). Postoperatively, LBP VAS score was significantly worse in the high-CSI group. Relative to preoperatively, postoperative leg pain and leg numbness improved significantly in both groups.

**Conclusions:**

We believe that neuro decompression can be effective for LSS surgical outcomes in patients with CS; nonetheless, it should be approached with caution owing to the potential for worsening LBP. Additionally, visceral fat is an important indicator suggesting the involvement of CS.

## Background

Lumbar spinal stenosis (LSS) is a degenerative condition involving spinal canal narrowing due to facet joint osteoarthritis, ligamentum flavum hypertrophy, intervertebral disc bulging, and spondylolisthesis [1]. Amundsen et al. [2, 3] reported that the most common symptoms in patients with LSS were back pain, including low back pain (LBP) (prevalence, 95%), claudication (91%), leg pain (71%), weakness (33%), and voiding disturbances (12%). When conservative treatments are less effective, surgical treatments, including decompression of neural tissues with or without fusion, show good clinical results [3, 4]. However, 20–40% of patients undergoing LSS surgery have poor outcomes, complaining of residual LBP, leg symptoms and gait disturbance [5–7]. A systematic review demonstrated that depression, cardiovascular comorbidity, disorder influencing walking ability, and scoliosis predicted poorer subjective outcomes [8]. However, the predictors of surgical outcomes for LSS remain controversial.

Obesity is associated with a higher degree of dissatisfaction and poorer outcomes after surgery for LSS, and patients with morbid obesity experience more complications than those with lower body mass index (BMI) [4, 9]. Additionally, obesity is commonly associated with larger amounts of visceral fat, subcutaneous fat, and abdominal circumference. Among these, visceral fat can induce systemic inflammation [10] and is associated with an increased risk of musculoskeletal and widespread pain, providing rationale for future research [11].

Central sensitization (CS) has recently been recognized as a potential pathophysiological cause of several chronic pain disorders, including chronic LBP (CLBP), chronic neck pain, myofascial pain syndrome, fibromyalgia, temporomandibular joint disorder, irritable bowel syndrome, interstitial cystitis, and tension-type headache [12–16]. These disorders have been classified on the mental-physical health spectrum as psychosomatic, medically unexplained, or due to functional or somatic factors [12, 14, 15]. CS involves an increased responsiveness of the central and/or peripheral nervous system circuits [12] and has been associated with chronic pain development [13]. The CS inventory (CSI), a patient-reported measure, has been widely used to evaluate the severity of CS and its related symptoms [17, 18]. Higher preoperative CS, evaluated using the CSI, has been associated with significantly worse surgical outcomes, including neurological symptoms, disability, and quality of life (QOL), especially related to LBP and psychological factors [19]. We previously reported that patients with a high CSI (≥ 40) had significantly higher visceral fat and LBP visual analogue scale (VAS) scores than those with a low CSI (< 40) [20]. Moreover, the visceral fat area had a moderately positive correlation with LBP VAS scores among patients with high CSI scores [20].

Therefore, to ascertain the role of visceral fat and CS in LSS, this study aimed to examine the relationship between CS, visceral fat area, and surgical outcomes in patients following surgery for LSS.

## Methods

The Institutional Review Board of Sapporo Medical University approved this study (IRB approval no. 302-1203). All participants were provided with written and verbal explanations of the study, and their consent was obtained prior to participation.

### Participants

We enrolled consecutive patients with LSS aged between 41 and 79 years. The surgeons performed physical testing on consecutive patients who reported with symptoms induced or exacerbated with walking or prolonged standing and relieved with lumbar flexion, sitting, and recumbency. These symptoms included pain, numbness, and neurological deficits in the lower extremities and buttocks as well as bladder or bowel dysfunction. Subsequently, radiographic and magnetic resonance imaging (MRI) findings suggestive of degenerative stenosis of the spinal canal or intervertebral foramen were correlated with the reported symptoms and clinical findings, thereby confirming the diagnosis of LSS. The same surgeons made the final diagnosis of symptomatic LSS, which necessitated the presentation both of clinical symptoms and radiographic findings of LSS. Symptoms of leg pain/numbness and intermittent claudication in patients with LSS who did not respond to conservative therapies for more than 3 months were considered to be indications for decompression surgery [21]. The exclusion criteria were as follows: age < 40 and > 80 years, acute trauma, infection, neoplasm, history of spinal surgery or spinal fracture, leg symptoms presentation for < 3 months, and spondylolisthesis with obvious intervertebral instability, identified as a pain generator causing LBP that may be improved by fusion surgery [22]. Intervertebral instability was defined as sagittal translation of > 3 mm, segmental motion of > 20°, or a posterior opening of > 5° on flexion/extension radiographs [23]. All participants rated their LBP, leg pain, and leg numbness using a VAS (0–100 mm). VASs are commonly used assessment tool for musculoskeletal pain intensity and have been proven reliable and valid [24]. Spinous process splitting laminectomy was performed as decompression surgery [25]. The participants completed clinical outcome assessments preoperatively and 12 months postoperatively. Finally, 60 patients were enrolled in the study (28 men and 32 women; mean age: 62.1 ± 2.8 years).

### Central sensitization evaluation

The CSI measures the extent to which an individual’s symptoms are likely attributable to CS [17, 18]. Part A comprises 25 symptom-related items scored on a 5-point Likert scale (0–4 for each item; total score range of 0–100). Part B identified patients with concurrent fibromyalgia. The CSI has been established as valid and reliable [17] with test–retest reliability of 0.82, Cronbach’s alpha of 0.88, sensitivity of 81%, and specificity of 75% [18]. The CSI has also been translated into Japanese and validated [26]. Neblett et al. [18] investigated patients referred to a multidisciplinary pain centre, specializing in the assessment and treatment of chronic pain, including central sensitivity syndromes (CSSs), such as fibromyalgia, chronic fatigue, and irritable bowel, for which CS may be a common aetiology. A receiver operating characteristic analysis determined that a CSI score of 40 out of 100 best distinguished patients with CSS from their non-patient comparators (area under the curve = 0.86, sensitivity = 81%, specificity = 75%). Thus, a cut-off score of 40 was used to identify low- and high-CS symptoms [27]. Therefore, in the present study, we divided the participants into low- (CSI < 40) and high- (CSI ≥ 40) CSI subgroups. CSI and VAS score measurements were conducted at the same time points for all patients.

### Computed tomography imaging

**Fig. 1:**
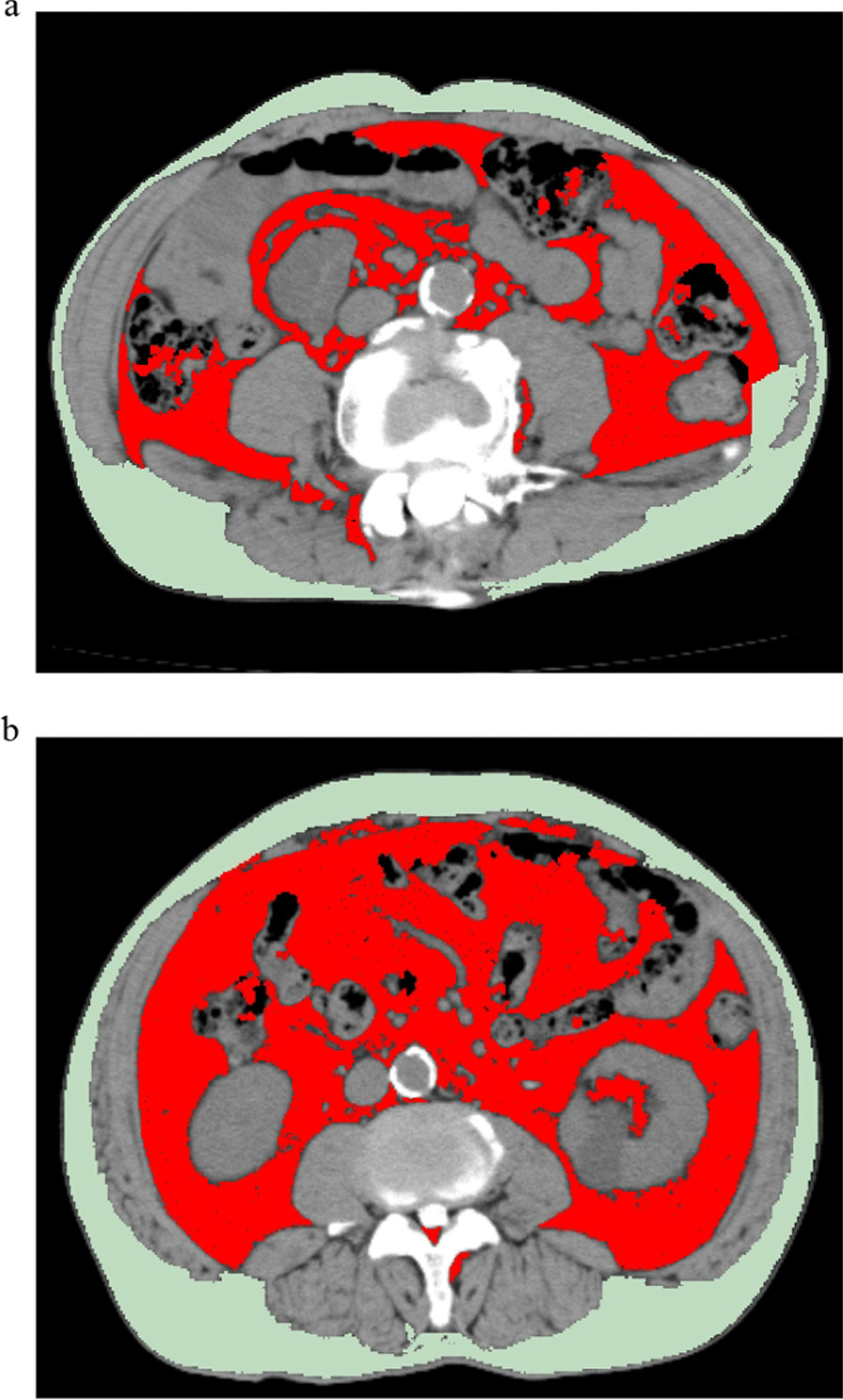
Abdominal CT images showing fat measurements (red: visceral fat; green: subcutaneous fat) in patients with low CSI scores (**a**) and high CSI scores (**b**). CT, computed tomography; CSI, central sensitization inventory

Visceral and subcutaneous fat cross-sectional areas were calculated semiautomatically using tissue-specific attenuation thresholds including adipose tissue (− 190 to − 30 Hounsfield unit [HU]) and skeletal muscle (− 29 to 150 HU) [28, 29]. Lee et al. [30] demonstrated excellent intra- and inter-observer reproducibility of adipose tissue measurements on CT images and reported the intra- and inter-observer agreements for the volume of visceral fat (intraclass correlation coefficient [ICC], 0.998 and 0.999; 95% confidence interval [CI] 0.996–0.999 and 0.999–1.000, respectively) and subcutaneous fat (ICC for intra- and inter-observer agreements, 0.998 and 0.997; 95% CI 0.996–0.999 and 0.994–0.998, respectively). CT-measured abdominal circumference referred to the waist perimeter, defined as the circumferential length of the outer margin of abdominal skin on axial CT images. Joo et al. [31] showed that CT-measured abdominal circumference was closely correlated with manually-measured abdominal circumference (*r* = 0.919; 95% CI 0.908–0.930], *p* < 0.001), and the ICC of CT-measured and manually-measured abdominal circumference was 0.954 (95% CI 0.947–0.960). The patients underwent CT imaging (Aquilion, Toshiba, Japan), which was performed simultaneously with myelography. We measured the visceral fat area, subcutaneous fat area, and abdominal circumference at the level of the umbilicus. The respiratory phase was at the end of exhalation, and the parameters were set as follows: tube voltage, 120 kV; tube current, 360 Ma; spin time, 0.5 s/rotation; and slice thickness, 0.6 mm. Typical CT images of a low-CSI patient and a high-CSI patient are presented in Fig. 1a and b, respectively.

### Statistical analyses

All numerical data are expressed as the mean ± standard error of the mean. Differences between men and women were analysed using the chi-square test. Additionally, differences between the groups regarding age, BMI, and VAS scores were compared using the Mann–Whitney *U* test. Analysis of covariance (ANCOVA) was used to compare the CT measurements between the low- and high-CSI groups, adjusted for age and sex. Differences in VAS scores between the pre- and post-12 months periods were analysed using the Mann–Whitney *U* test. All statistical analyses were performed using SPSS (version 27.0; IBM Corp., Armonk, NY, USA). Statistical significance was set at *p* < 0.05.

## Results

**Fig. 2:**
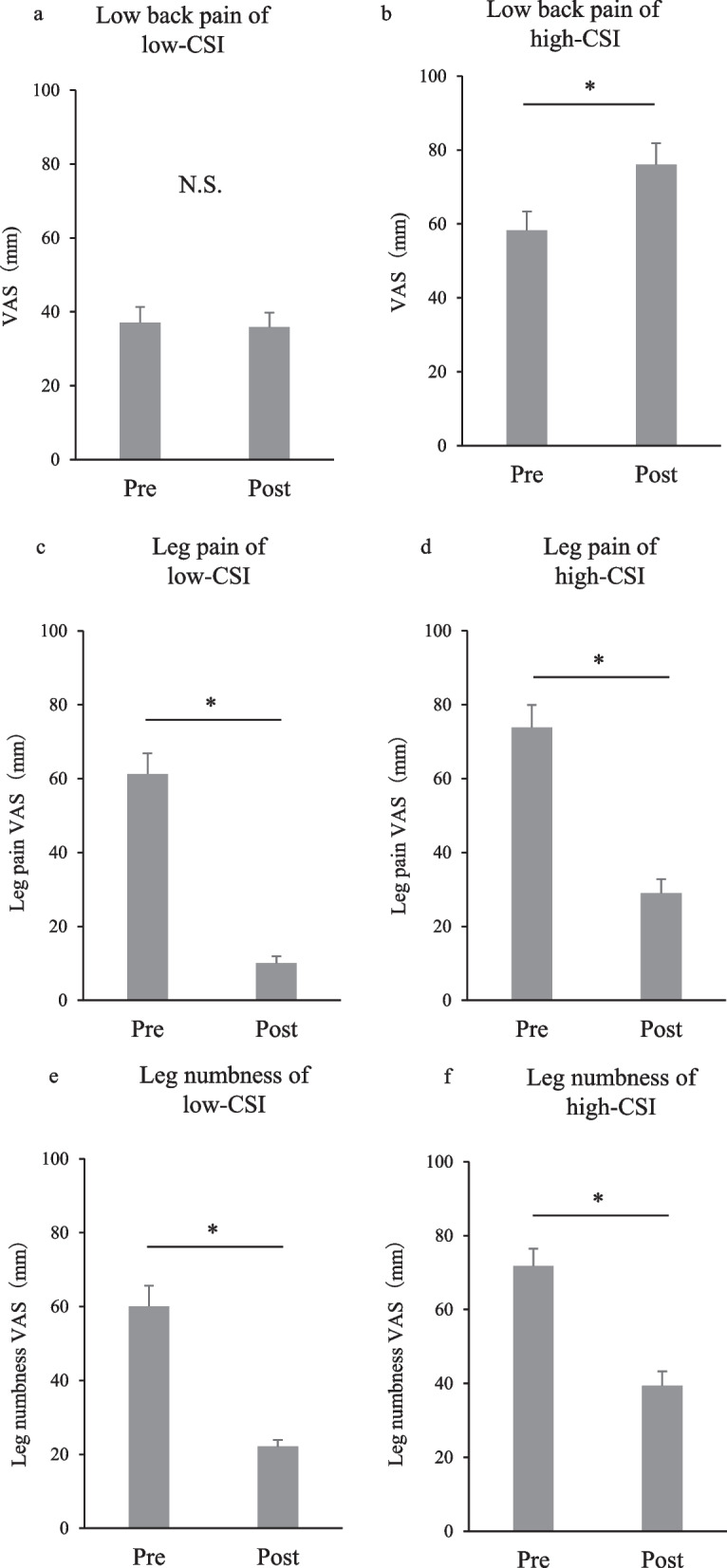
Comparison of preoperative and postoperative low back pain (**a**), leg pain (**b**), and leg numbness (**c**) in the high-CSI and low-CSI groups. LBP, low back pain; CSI, central sensitization inventory; VAS, visual analogue scale

**Fig. 3:**
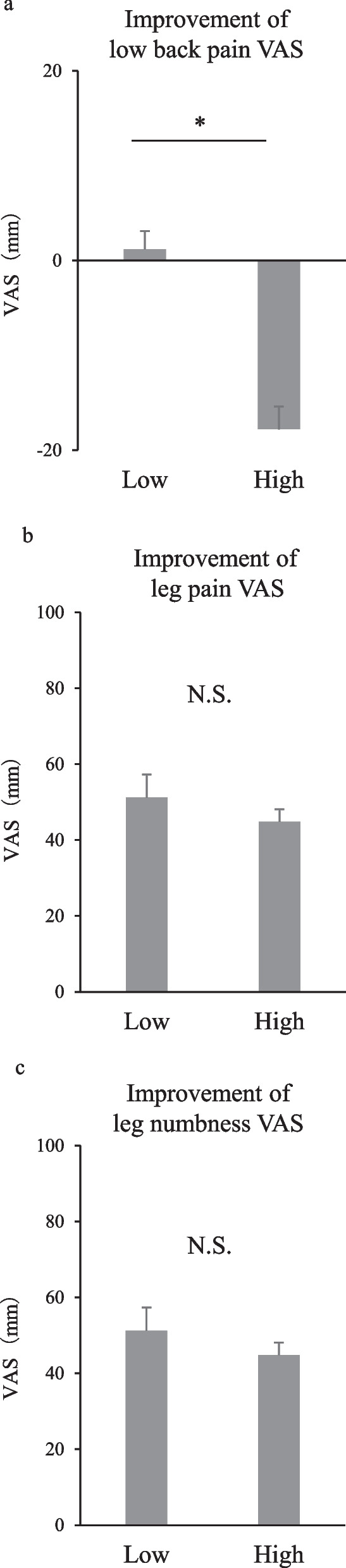
Comparison of low back pain (**a**), leg pain (**b**), and leg numbness (**c**) improvement in the high-CSI and low-CSI groups. CSI, central sensitization inventory; VAS, visual analogue scale

As shown in Table 1, the low-CSI group comprised 39 patients (65.0%; 19 men, 21 women), and the high-CSI group comprised 21 patients (35.0%; 9 men, 11 women). The difference in mean BMI between the two groups was not statistically significant (*p* = 0.72). The high-CSI group had significantly higher mean LBP, leg pain, and leg numbness VAS score compared to the low-CSI group (*p* < 0.01). Table 2 shows that the high-CSI group had a significantly higher mean visceral fat area than that of the low-CSI group (*p* < 0.01). No statistically significant differences were found in the mean subcutaneous fat area (*p* = 0.65) or abdominal circumference (*p* = 0.41) between the two groups. The mean preoperative and postoperative LBP VAS scores, respectively, were 37.1 ± 4.2 and 35.9 ± 3.9 mm in the low-CSI group (Fig. 2a), and 58.3 ± 5.1 and 76.1 ± 5.8 mm in the high-CSI group (Fig. 2b). The treatment outcome of LBP VAS was significantly worse in the high-CSI group (Fig. 2b). The improvement of LBP VAS was significantly worse in the high-CSI group compared to the low-CSI group (Fig. 3a). The mean preoperative and postoperative leg pain VAS scores, respectively, were 61.3 ± 5.6 and 10.1 ± 1.8 mm in the low-CSI group (Fig. 2c), and 73.8 ± 6.1 and 29.0 ± 3.8 mm in the high-CSI group (Fig. 2d). The mean preoperative and postoperative VAS scores for mean leg numbness were 60.1 ± 4.5 and 22.1 ± 3.1 mm in the low-CSI group (Fig. 2e) and 71.8 ± 4.7 and 39.4 ± 3.9 mm in the high-CSI group, respectively (Fig. 2f). Leg pain and leg numbness improved significantly in both groups. There was no significant difference in the improvement of leg pain and numbness between high-CSI and low-CSI groups (Fig. 3b, c).

**Table 1:** Demographic data of patients in the low- and high-CSI groups

	Low CSI	High CSI	*p* value
Sex (M: W)	19:21	9:11	0.85*
Age (years)	63.1 ± 2.3	61.7 ± 1.6	0.46**
BMI (kg/m^2^)	23.1 ± 0.6	24.6 ± 1.0	0.72**
LBP VAS (mm)	37.1 ± 4.2	58.3 ± 5.1	< 0.01**
Leg pain VAS (mm)	61.3 ± 5.6	73.8 ± 6.1	< 0.01**
Leg numbness VAS (mm)	60.1 ± 4.5	71.8 ± 4.7	< 0.01**

Data are expressed as the mean ± standard error of the mean

BMI, body mass index; VAS, visual analogue scale; CSI, central sensitization inventory; M, men; W, women

*Chi-square test

**Mann–Whitney *U*-test

**Table 2:** Comparisons of CT measurements in the low- and high-CSI groups using analysis of covariance adjusted for age and sex

	Low CSI	High CSI	*p* value
Visceral fat area (cm^2^)	135.2 ± 6.6	165.2 ± 9.9	< 0.01*
Subcutaneous fat area (cm^2^)	138.1 ± 6.2	143.1 ± 8.7	0.65*
Abdominal circumference (cm^2^)	84.1 ± 2.3	88.0 ± 2.7	0.41*

Data are expressed as the estimated mean ± standard error of the mean

CT, computed tomography; CSI, central sensitization inventory

*Analysis of covariance adjusted for age and sex

## Discussion

In the present study, the prevalence of high-CSI was 35.0% (high-CSI, *n* = 21; low-CSI, *n* = 39). A previous study involving patients with chronic spinal pain disorder with musculoskeletal injury reported that 67% of the patients were classified into the high-CSI group upon admission for an interdisciplinary functional restoration programme [32]. Tanaka et al. [33] reported that among 553 patients with musculoskeletal disorders in a primary care setting, 24.4% had a high CSI. Additionally, Mibu et al. [34] reported that 18.3% of patients with CLBP from two orthopaedic clinics were diagnosed with CS. Although the patient characteristics varied among these studies, we considered chronic pain accompanied by CS as similar cases.

There were two important findings concerning the preoperative analysis in the current study. First, the high-CSI group had significantly higher VAS scores than those of the low-CSI group, suggesting that patients with high-CSI experienced more severe LBP, leg pain, and leg numbness. In a previous report, pain severity based on CSI score was significantly related to other types of pain intensity, pain-related anxiety, depressive symptoms, somatization symptoms, perceived disability, and sleep disturbance [32]. Patients with CLBP experienced more severe pain at equal pressure levels, and functional MRIs revealed more widespread patterns of neuronal activation in pain-related cortical areas [35]. Mibu et al. [34] found that patients with CLBP exhibited more pronounced CS-related symptoms than patients with knee osteoarthritis, and the CSI scores in patients with CLBP were associated with pain-related disability and health-related quality of life. CSI is significantly associated with preoperative neurological symptoms and the health-related quality of life of patients who undergo lumbar spine surgeries [36]. Second, the high-CSI group had significantly more visceral fat compared to the low-CSI group. One possible explanation relates to visceral fat promoting chronic low-grade systemic inflammation [10, 11]; however, the mechanisms underlying the association between visceral fat and CS are not yet well understood. We posit that first understanding the association between visceral fat and CS is important for gaining valuable insights. These two findings indicated that CLBP, leg pain, and leg numbness should be included in the list of conditions associated with visceral fat accumulation. To our knowledge, no previous study has explored the association of visceral fat and CS, which may contribute to CLBP, leg pain, and leg numbness intensity. Thus, we believe our study may provide new insights into the assessment of CLBP, leg pain, and leg numbness in patients with LSS.

In the present study, the high-CSI group presented with more symptoms and reported higher LBP intensity after lumber decompression relative to before surgery. A high preoperative CSI score is associated with worse quality of life and increased duration of hospital stay following spinal fusion [37]. Moreover, recent evidence indicated that CS is associated with more severe total knee arthroplasty postoperative pain and diminished patient satisfaction [38]. The conditions characterized by recurring LBP after spine surgery is termed failed back surgery syndrome (FBSS) [39]. FBSS incidence ranges between 10 and 40% after lumbar laminectomy with or without fusion [40]. Patient psychological factors such as anxiety, depression, and hypochondriasis or social characteristics, such as economic status and litigation, may contribute to the aetiology of FBSS [40]. In addition to these factors, CS may also be a factor in FBSS. In contrast, leg pain and leg numbness improved in both groups in the present study, although the improvement in the high-CSI group was significantly greater than that in the low-CSI group. Akeda et al. [19] revealed that patients with LSS, whose preoperative CSI score was ≥ 40, are expected to have worse neurological symptoms, disability, and quality of life compared to those with a CSI score of < 40. However, postoperative leg pain and leg numbness improved relative to before surgery. This previous report that analysed surgical outcomes of surgery for LSS and CS also supports our current results.

The outcomes of our study indicate that neuro decompression can be effective for LSS with CS; nevertheless, the caveat of the potential for worsened LBP must be considered. In addition, our study highlights the importance of visceral fat in the involvement of CS. To this end, under the condition of giving informed consent before surgery, surgeons should explain the factors influencing postoperative improvement of lower extremity symptoms and worsening of LBP to patients with a high-CSI, ensure patients’ understanding of the expected postoperative recovery outcomes, and avoid patients’ insufficient understanding of heightened LBP postoperatively, thereby improving postoperative satisfaction.

This study has some limitations. First, this study had a relatively short follow-up period of 12 months; postoperative outcomes might change over a longer periods. Second, the duration of neurological symptoms from onset to the lumbar surgeries may affect the extent of CS pre- and postoperatively; however, this was not evaluated in this study. In the future, we would like to investigate whether preoperative interventions, such as aerobic exercise and medical diet, can improve CS along with visceral fat reduction. Subsequently, we would endower to compare surgical outcomes concerning LBP between patients receiving a preoperative aerobic exercise and medical diet interventions and those receiving no intervention. We believe this would contribute to understanding the pathogenesis of LBP after LSS surgery and improve surgical outcomes.

## Conclusions

This study evaluated the relationship between CS, visceral fat area, and surgical outcomes in LSS. We report that patients with a high CSI had significantly higher VAS scores, indicative of more severe LBP, leg pain, and leg numbness, than those with a low-CSI. Additionally, patients with a high CSI had significantly more visceral fat compared to those with low CSI, implying the role of visceral fat in CS. Furthermore, patients with a high CSI presented with more symptoms and reported higher LBP intensity after lumber decompression than before surgery, while leg pain and leg numbness improved in both groups, albeit to a greater extent in patients with a lower preoperative CSI. We conclude that neuro decompression can be effective for LSS with CS; nevertheless, caution must be taken for the possibility of worsened postoperative LBP.

## Data Availability

The datasets generated and analysed during the current study are not publicly available due to limitations of ethical approval involving the patient data and anonymity but are available from the corresponding author on reasonable request.

## Abbreviations

BMI: Body mass index
CLBP: Chronic low back pain
CSI: Central sensitization inventory
CS: Central sensitization
FBSS: Failed back surgery syndrome
ICC: Intraclass correlation coefficient
LBP: Low back pain
LSS: Lumbar spinal stenosis
VAS: Visual analogue scale

## References

[1] Katz JN, Harris MB (2008). Clinical practice. Lumbar spinal stenosis. N Engl J Med.

[2] Amundsen T, Weber H, Lilleås F, Nordal HJ, Abdelnoor M, Magnaes B (1995). Lumbar spinal stenosis. Clinical and radiologic features. Spine (Phila Pa 1976).

[3] Amundsen T, Weber H, Nordal HJ, Magnaes B, Abdelnoor M, Lilleâs F (2000). Lumbar spinal stenosis: conservative or surgical management?: A prospective 10-year study. Spine (Phila Pa 1976).

[4] Katz JN, Zimmerman ZE, Mass H, Makhni MC (2022). Diagnosis and management of lumbar spinal stenosis: a review. JAMA.

[5] Xia Y, Ishii K, Matsumoto M, Namamura M, Toyama Y, Chiba K (2008). Radiographic predictors of residual low back pain after laminectomy for lumbar spinal canal stenosis: minimum 5-year follow-up. J Spinal Disord Tech.

[6] Katz JN, Stucki G, Lipson SJ, Fossel AH, Grobler LJ, Weinstein JN (1999). Predictors of surgical outcome in degenerative lumbar spinal stenosis. Spine (Phila Pa 1976).

[7] Hara N, Oka H, Yamazaki T, Takeshita K, Murakami M, Hoshi K (2010). Predictors of residual symptoms in lower extremities after decompression surgery on lumbar spinal stenosis. Eur Spine J.

[8] Aalto TJ, Malmivaara A, Kovacs F, Herno A, Alen M, Salmi L (2006). Preoperative predictors for postoperative clinical outcome in lumbar spinal stenosis: systematic review. Spine (Phila Pa 1976).

[9] Hareni N, Gudlaugsson K, Strömqvist F, Rosengren BE, Karlsson MK (2022). A comparison study on patient-reported outcome between obese and non-obese patients with central lumbar spinal stenosis undergoing surgical decompression: 14,984 patients in the National Swedish Quality Registry for Spine Surgery. Acta Orthop.

[10] Shin J, Syme C, Wang D, Richer L, Pike GB, Gaudet D (2019). Novel genetic locus of visceral fat and systemic inflammation. J Clin Endocrinol Metab.

[11] Li S, Schwartz AV, LaValley MP, Wang N, Desai N, Sun X (2020). Association of visceral adiposity with pain but not structural osteoarthritis. Arthritis Rheumatol.

[12] Woolf CJ (2011). Central sensitization: Implications for the diagnosis and treatment of pain. Pain.

[13] Sanzarello I, Merlini L, Rosa MA, Perrone M, Frugiuele J, Borghi R (2016). Central sensitization in chronic low back pain: a narrative review. J Back Musculoskelet Rehabil.

[14] Smart KM, Blake C, Staines A, Thacker M, Doody C (2012). Mechanisms-based classifications of musculoskeletal pain: part 1 of 3: symptoms and signs of central sensitisation in patients with low back (+/- leg) pain. Man Ther.

[15] Petersel DL, Dror V, Cheung R (2011). Central amplification and fibromyalgia: disorder of pain processing. J Neurosci Res.

[16] Nijs J, Apeldoorn A, Hallegraeff H, Clark J, Smeets R, Malfliet A (2015). Low back pain: guidelines for the clinical classification of predominant neuropathic, nociceptive, or central sensitization pain. Pain Physician.

[17] Mayer TG, Neblett R, Cohen H, Howard KJ, Choi YH, Williams MJ (2012). The Development and psychometric validation of the central sensitization inventory. Pain Pract.

[18] Neblett R, Cohen H, Choi Y, Hartzell MM, Williams M, Mayer TG (2013). The central sensitization inventory (CSI): establishing clinically significant values for identifying central sensitivity syndromes in an outpatient chronic pain sample. J Pain.

[19] Akeda K, Yamada J, Takegami N, Fujiwara T, Murata K, Kono T (2023). Central sensitization as a predictive factor for the surgical outcome in patients with lumbar spinal stenosis: a multicenter prospective study. Eur Spine J.

[20] Ogon I, Teramoto A, Takashima H, Terashima Y, Yoshimoto M, Emori M (2022). Associations between visceral fat chronic low back pain and central sensitization in patients with lumbar spinal stenosis. J Back Musculoskelet Rehabil.

[21] Kreiner DS, Shaffer WO, Baisden JL, Gilbert TJ, Summers JT, Toton JF (2013). An evidence-based clinical guideline for the diagnosis and treatment of degenerative lumbar spinal stenosis (update). Spine J.

[22] Hauser RA, Matias D, Woznica D, Rawlings B, Woldin BA (2022). Lumbar instability as an etiology of low back pain and its treatment by prolotherapy: a review. J Back Musculoskelet Rehabil.

[23] Yoshimoto M, Miyakawa T, Takebayashi T, Ida K, Tanimoto K, Kawamura S (2014). Microendoscopy-assisted muscle-preserving interlaminar decompression for lumbar spinal stenosis: clinical results of consecutive 105 cases with more than 3-year follow-up. Spine (Phila Pa 1976).

[24] Crossley KM, Bennell KL, Cowan SM, Green S (2004). Analysis of outcome measures for persons with patellofemoral pain: which are reliable and valid?. Arch Phys Med Rehabil.

[25] Watanabe K, Hosoya T, Shiraishi T, Matsumoto M, Chiba K, Toyama Y (2005). Lumbar spinous process-splitting laminectomy for lumbar canal stenosis. Technical note. J Neurosurg Spine.

[26] Tanaka K, Nishigami T, Mibu A, Manfuku M, Yono S, Shinohara Y (2017). Validation of the Japanese version of the central sensitization inventory in patients with musculoskeletal disorders. PLoS ONE.

[27] Neblett R, Hartzell MM, Mayer TG, Cohen H, Gatchel RJ (2017). Establishing clinically relevant severity levels for the central sensitization inventory. Pain Pract.

[28] van Vugt JL, Levolger S, Gharbharan A, Koek M, Niessen WJ, Burger JW (2017). A comparative study of software programmes for cross-sectional skeletal muscle and adipose tissue measurements on abdominal computed tomography scans of rectal cancer patients. J Cachexia Sarcopenia Muscle.

[29] Borrelli P, Kaboteh R, Enqvist O, Ulén J, Trägårdh E, Kjölhede H (2021). Artificial intelligence-aided CT segmentation for body composition analysis: a validation study. Eur Radiol Exp.

[30] Lee EJ, Cho NJ, Kim H, Nam B, Jeon JS, Noh H (2022). Abdominal periaortic and renal sinus fat attenuation indices measured on computed tomography are associated with metabolic syndrome. Eur Radiol.

[31] Joo I, Kwak MS, Park DH, Yoon SH (2021). Fully automated waist circumference measurement on abdominal CT: comparison with manual measurements and potential value for identifying overweight and obesity as an adjunct output of CT scan. PLoS ONE.

[32] Neblett R, Hartzell MM, Williams M, Bevers KR, Mayer TG, Gatchel RJ (2017). Use of the central sensitization inventory (CSI) as a treatment outcome measure for patients with chronic spinal pain disorder in a functional restoration program. Spine J.

[33] Tanaka K, Murata S, Nishigami T, Mibu A, Manfuku M, Shinohara Y (2019). The central sensitization inventory predict pain-related disability for musculoskeletal disorders in the primary care setting. Eur J Pain.

[34] Mibu A, Nishigami T, Tanaka K, Manfuku M, Yono S (2019). Difference in the impact of central sensitization on pain-related symptoms between patients with chronic low back pain and knee osteoarthritis. J Pain Res.

[35] Giesecke T, Gracely RH, Grant MA, Nachemson A, Petzke F, Williams DA (2004). Evidence of augmented central pain processing in idiopathic chronic low back pain. Arthritis Rheum.

[36] Akeda K, Yamada J, Takegami N, Fujiwara T, Murata K, Kono T (2023). Evaluation of central sensitization inventory in patients undergoing elective spine surgery in a multicenter study. Glob Spine J.

[37] Bennett EE, Walsh KM, Thompson NR, Krishnaney AA (2017). Central sensitization inventory as a predictor of worse quality of life measures and increased length of stay following spinal fusion. World Neurosurg.

[38] Kim SH, Yoon KB, Yoon DM, Yoo JH, Ahn KR (2015). Influence of centrally mediated symptoms on postoperative pain in osteoarthritis patients undergoing total knee arthroplasty: a prospective observational evaluation. Pain Pract.

[39] Kregel J, Schumacher C, Dolphens M, Malfliet A, Goubert D, Lenoir D (2018). Convergent validity of the Dutch central sensitization inventory: associations with psychophysical pain measures, quality of life, disability, and pain cognitions in patients with chronic spinal pain. Pain Pract.

[40] Chan CW, Peng P (2011). Failed back surgery syndrome. Pain Med.

